# Vitamin D modulation of brain-gut-virome disorder caused by polystyrene nanoplastics exposure in zebrafish (*Danio rerio*)

**DOI:** 10.1186/s40168-023-01680-1

**Published:** 2023-11-27

**Authors:** Miaomiao Teng, Yunxia Li, Xiaoli Zhao, Jason C. White, Lihui Zhao, Jiaqi Sun, Wentao Zhu, Fengchang Wu

**Affiliations:** 1 State Key Laboratory of Environmental Criteria and Risk Assessment, Chinese Research Academy of Environmental Sciences, Beijing, 100012 China; 2 The Connecticut Agricultural Experiment Station, New Haven, CT 06511 USA; 3 School of Energy and Environmental Engineering, University of Science and Technology Beijing, Beijing, 100083 China; 4 Department of Applied Chemistry, Innovation Center of Pesticide Research, College of Science, China Agricultural University, Beijing, 100193 China

**Keywords:** Polystyrene nanoplastics, Vitamin D, Brain, Viruses, Zebrafish

## Abstract

**Background:**

Many studies have investigated how nanoplastics (NPs) exposure mediates nerve and intestinal toxicity through a dysregulated brain-gut axis interaction, but there are few studies aimed at alleviating those effects. To determine whether and how vitamin D can impact that toxicity, fish were supplemented with a vitamin D-low diet and vitamin D-high diet.

**Results:**

Transmission electron microscopy (TEM) showed that polystyrene nanoplastics (PS-NPs) accumulated in zebrafish brain and intestine, resulting in brain blood–brain barrier basement membrane damage and the vacuolization of intestinal goblet cells and mitochondria. A high concentration of vitamin D reduced the accumulation of PS-NPs in zebrafish brain tissues by 20% and intestinal tissues by 58.8% and 52.2%, respectively, and alleviated the pathological damage induced by PS-NPs. Adequate vitamin D significantly increased the content of serotonin (5-HT) and reduced the anxiety-like behavior of zebrafish caused by PS-NPs exposure. Virus metagenome showed that PS-NPs exposure affected the composition and abundance of zebrafish intestinal viruses. Differentially expressed viruses in the vitamin D-low and vitamin D-high group affected the secretion of brain neurotransmitters in zebrafish. Virus *AF191073* was negatively correlated with neurotransmitter 5-HT, whereas *KT319643* was positively correlated with malondialdehyde (MDA) content and the expression of cytochrome 1a1 (*cyp1a1*) and cytochrome 1b1 (*cyp1b1*) in the intestine. This suggests that *AF191073* and *KT319643* may be key viruses that mediate the vitamin D reduction in neurotoxicity and immunotoxicity induced by PS-NPs.

**Conclusion:**

Vitamin D can alleviate neurotoxicity and immunotoxicity induced by PS-NPs exposure by directionally altering the gut virome. These findings highlight the potential of vitamin D to alleviate the brain-gut-virome disorder caused by PS-NPs exposure and suggest potential therapeutic strategies to reduce the risk of NPs toxicity in aquaculture, that is, adding adequate vitamin D to diet.

Video Abstract

**Supplementary Information:**

The online version contains supplementary material available at 10.1186/s40168-023-01680-1.

## Background

Plastics are indispensable in daily life, with rates of production continuing to increase, but the recovery rate in many countries is quite low; for example, in China, South Korea, and Australia, the recovery is only 25%, 13.5%, and 11.5%, respectively [1, 2]. It is estimated that by 2030, without effective waste management methods, 9 × 10^7^ t of plastic waste will enter aquatic ecosystems every year, seriously threatening the survival of aquatic species [3]. After a long-term physical, chemical, and biological reactions, plastics will degrade into microplastics (*MPs*, < 5 mm) and eventually nanoplastics (*NPs*, < 100 nm) [4]. At present, MPs/NPs have been detected in numerous aquatic environments. The abundance of MPs/NPs in typical inflow rivers of Taihu Lake is 160–700 items/m^3^ [5]. The high abundant of MPs/NPs in surface water may compromise the drinking water safety. MPs/NPs have been detected in drinking water treatment plants (DWTP) near Barcelona, with abundance from 4230 ± 1260 to 75 ± 19 items/m^3^ in the influent and effluent of the plant, respectively [6]. In addition, MPs/NPs with different numbers and shapes have been detected in edible salt and seafood in many countries, demonstrating contaminant entry into the food chain and may pose unknown threat to human health [7, 8]. It has been reported that MPs/NPs with specific size and shape characteristics can be ingested by aquatic animal species, and that the residual MPs/NPs may induce toxicity, such as oxidative stress, immune injury, lipid membrane damage, and reproductive toxicity [9–11]. Teng et al. exposed zebrafish to 1, 10, and 100 μg/L polystyrene nanoplastics (PS-NPs) (44 nm) for 30 days and found that PS-NPs inhibited growth and adversely impacted inflammatory response and intestinal permeability [12]. It is worth noting that most published studies investigating the toxicity and bioaccumulation of MPs/NPs in aquatic organisms have been conducted under laboratory conditions at concentrations that far exceed that of environmental relevance. For example, Murphy et al. used MPs/NPs concentrations of 1 × 10^7^, 2 × 10^7^, 4 × 10^7^, and 8 × 10^7^ μg/L to explore the effects of MPs/NPs on the feeding, morphology, and reproduction of *Hydra attenuate*, which far exceeded the environmental concentration [13]. Lenz et al. stated that MPs/NPs exposure studies must be at environmentally realistic concentrations to avoid misunderstanding the actual toxicity of the xenobiotics [14]. Al-Sid-Cheikh et al. studied the uptake, whole-body distribution, and depuration of NPs by scallops (*Pecten maximus*) at environmentally realistic concentrations (15 μg/L) and reported that 250-nm NPs accumulated in the intestine, while 24-nm NPs were dispersed throughout the body [15]. The authors noted that it could take 300 days of continued environmental exposure for uptake to reach equilibrium in scallop body tissues. Consequently, it is of great importance to conduct exposures at environmentally relevant concentrations to accurately understand the fate and effects of these materials in aquatic ecosystems.

At present, MPs/NPs are known to have potential effects on brain nerve cells and intestinal health, prompting a focus on brain neurotransmitters and the intestinal microbiome. Neurotransmitters are the main endogenous metabolites of nerve transmission and are the key components for regulating central nervous system (CNS) and peripheral nervous system (PNS) circuits and activity, including the gastrointestinal tract [16]. The intestinal microbiome has been shown to impact the secretion of several neurotransmitters, playing a key role in both nerve and cognitive regulation, neurological disorders, and forming the basis of the brain-gut-microbiome axis [17–19]. Teng et al. found that exposure to PS-NPs disrupted the brain-gut axis relationship of zebrafish, and that the potential mechanism of toxicity was mediated by the altered neurotransmitter metabolites and the response of the intestinal flora to PS-NPs exposure [12]. Huang et al. used Phylogenetic Investigation of Communities by Reconstruction of Unobserved States (PICRUST) to predict that PS-NPs may directly affect the secretion of neurotransmitters by interfering with the structure of fish intestinal flora, thereby establishing a new mechanism by which NPs might cause behavioral toxicity through the brain-gut-microbiota axis [20].

The intestinal microflora is composed of bacteria, viruses, and fungi [21]. Notably, much of the research on intestinal microorganisms focuses on bacteria while paying less attention to the role of viruses and fungi. The virome is one of the main forces that shapes intestinal microbiota, but it is also the most poorly understood [22]. Similar to the bacterial microflora, most endogenous viruses do not cause disease, which indicates that many may be involved with symbiotic or mutually beneficial interactions [23]. When the structure of the virus community composition is disrupted, a range of pathological changes can occur, such as inflammatory bowel disease (IBD). IBD is mainly characterized by intestinal inflammation and intestinal injury, which have been studied and manifested in fish [24]. In addition, studies have shown that the virome can regulate the brain-gut axis interaction. Seth et al. reported that altered virome diversity had a positive correlation with serum interleukin 6 (*IL-6*), interleukin-1 beta (*IL-1β*), and decreased brain-derived neurotrophic factor (BDNF), a neurogenesis marker, indicating that there was an interaction between brain-gut virome [25]. In addition, bacteriophages are the main component of the virome, playing a vital role in many microbial communities, and are now known to be an important component in the microbiome-brain axis [26]. Studies have revealed the virome composition in fish, but the influence of viruses abundance change on zebrafish has not been reported. Fortunately, the development of metagenomics greatly advanced efforts to understand the basic biology of virome. In the current study, we explore the relationship between the zebrafish gut virome and brain-gut axis by metagenomics, with the aim of revealing the role of virome in PS-NPs-induced neurotoxicity.

Notably, many substances have been used to aid in the detoxification of environmental contaminants, such as probiotics, phytochemicals, and vitamins. Chen et al. demonstrated the potential of probiotics in regulating intestinal microbial imbalance and lipid metabolism disorder caused by PFBS exposure [27]. Saha et al. showed that 100 mg/kg andrographolide could decrease microglial activation, increase neurotrophic factor BDNF, and alleviate the pathological changes of brain-gut axis induced by Gulf War chemicals (permethrin and pyridostigmine bromide) [28]. Moniruzzaman et al. showed that dietary supplementation of selenium, vitamin C, and vitamin E could reduce serum lipid peroxidation and promote survival after high Hg exposure in mice [29]. However, there is few research on the potential of vitamin D to alleviate xenobiotic toxicity. Vitamin D is a pleiotropic hormone that has intestinal functionality and has a wide range of effects on intestinal barrier function (such as the integrity of epithelial cells), immune regulation, and the gut microbiome [30, 31]. Liao et al. showed that the expression of antimicrobial peptides (AMPs) and interleukin 22 (*IL-22*) restrained, and the susceptibility to bacterial infection was increased in VD-deficient zebrafish. In addition, VD induced the expression of AMP in zebrafish intestine by activating *IL-22* signal transduction dependent on microbial population, thus fighting bacterial infection [32]. Vitamin D deficiency not only is related to various immune diseases but also is involved in response to viral infection [33]. Zhang et al. found that the virome profile and virome-bacterial interactions in the gut of mice changed significantly after deleting the vitamin D receptor (VDR) from intestinal epithelial cells, Paneth cells, and myeloid cells [34]. Considering the beneficial effects of vitamin D and its multi-target properties, we investigated whether the administration of vitamin D in PS-NPs-treated zebrafish can restore the altered virome pattern and reduce the toxic effects induced by PS-NPs.

In this study, PS-NPs-exposed zebrafish were subjected to a novel tank diving test to investigate potential behavioral toxicity. In addition, the relationship between the gut virome, brain neurotransmitters, and intestinal biochemical parameters was evaluated to understand the immune and neurotoxicity of PS-NPs at an environmentally relevant concentration. Zebrafish were also co-exposed to vitamin D-low diet and vitamin D-high diet to understand the potential to alleviate PS-NPs toxicity. The results of this study provide baseline data for the application of vitamin D in PS-NPs polluted areas and can be offered as a strategy to minimize toxicity in aquaculture operations.

## Results

### Effects of exposure to PS-NPs and vitamin D on zebrafish growth parameters

**Fig. 1:**
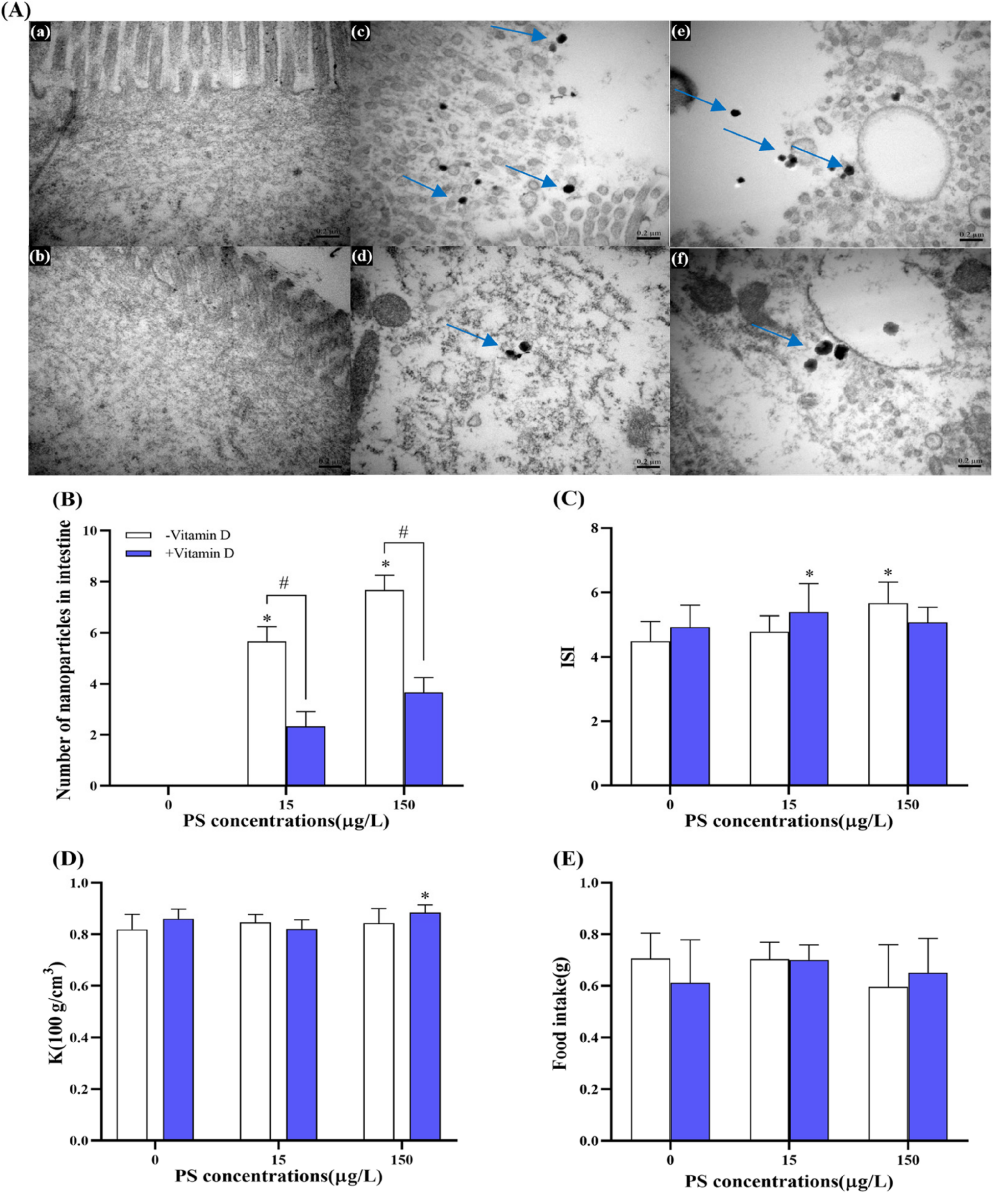
Accumulation of PS-NPs in the intestinal tissues of zebrafish and changes in related growth parameters. **A** Transmission electron microscopy (TEM) observation of PS-NPs in intestinal tissue (0.2 μm), a, b, c, d, e, and f represent the group of 0 − , 0 + , 15 − , 15 + , 150 − , and 150 + , respectively; the blue arrow points to the NPs. **B** The number of NPs in intestine tissue (*n* = 3 replicates). **C** ISI (%). **D** K (100 g/cm^3^). **E** FI (g). Data are expressed as means ± standard deviation (SD). *p < 0.05 indicate significant differences between exposure groups and the control group; #*p* < 0.05 indicate significant differences between vitamin D-high and vitamin D-low groups at the same PS-NPs concentration

After 21 days of exposure to PS-NPs, NPs were detected in the brain tissue. Compared with 15 − and 150 − , 15 + and 150 + groups both had reduced number of NPs by 20%, although there was no significant difference. Compared with the controls, the brain-somatic index (BSI) of zebrafish was slightly increased by PS-NPs exposure, but there was no significant difference, also between the vitamin D-low and vitamin D-high group (Figure S1). Different amounts of NPs accumulated in the intestinal tissues of zebrafish under PS-NPs, with a dose-dependent increase being evident (Fig. 1A, B). Notably, compared with 15 − and 150 − , 15 + and 150 + groups exhibited significantly reduced numbers of nanoparticles in the intestinal tract, by 58.8% and 52.2%, respectively (Fig. 1B). In addition, the intestine-somatic index (ISI) in 15 + and 150 − was significantly increased compared with controls (Fig. 1C). Food intake (FI) of zebrafish was unaffected by treatment (Fig. 1E). However, the K of 150 + increased significantly compared with controls (Fig. 1D).

### Histopathological changes of the brain and related parameters

**Fig. 2:**
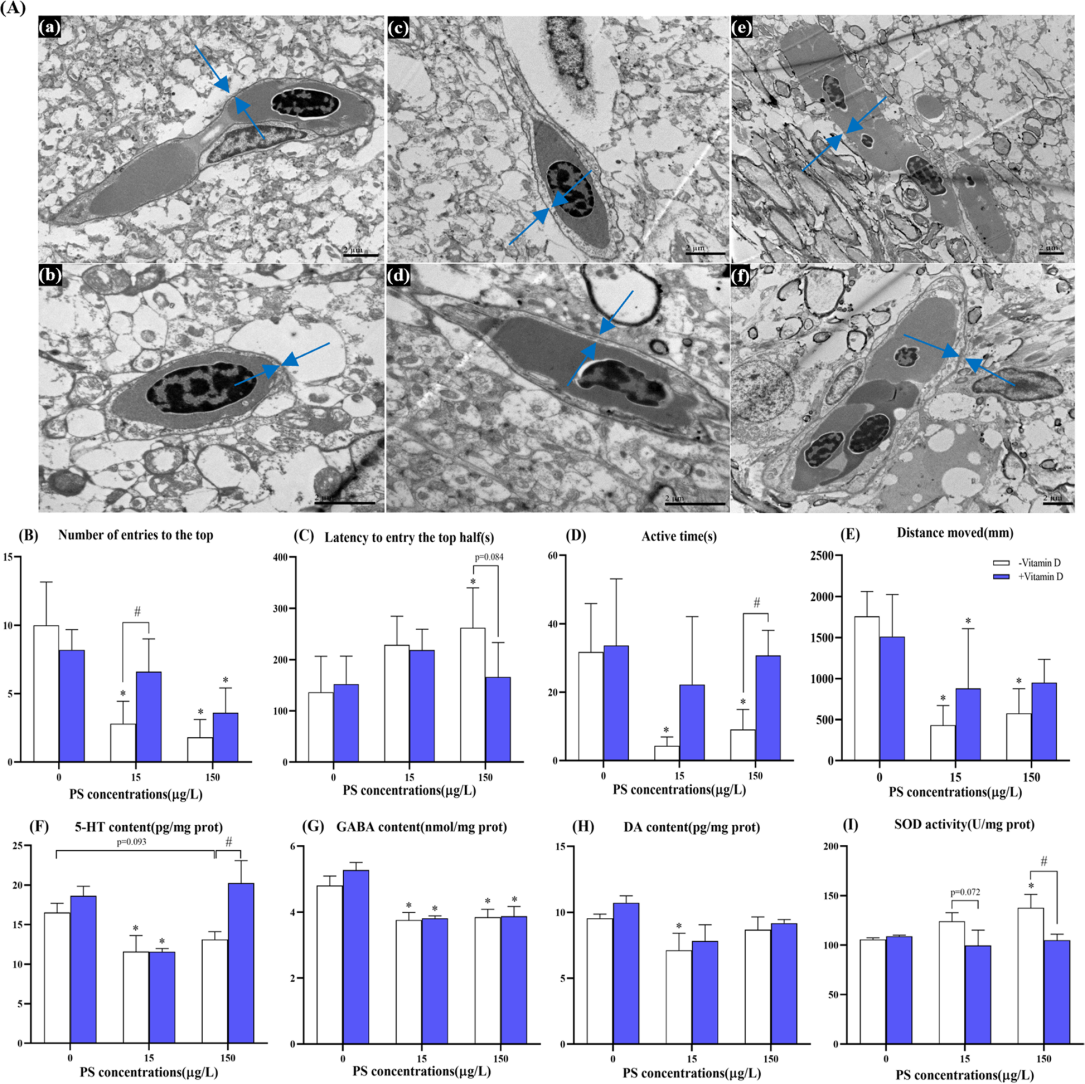
Representative histological changes of brain tissue samples and changes of related parameters. **A** TEM of the blood–brain barrier (2 μm), a, b, c, d, e, and f represent the group of 0 − , 0 + , 15 − , 15 + , 150 − , and 150 + , respectively. The blue arrow represents the basement membrane of the blood–brain barrier. **B** The number of entries to the top. **C** Latency to entry the top half. **D** Active time. **E** Distance moved. **F**, **G**, and **H** represent the content of 5-HT, GABA, and DA in zebrafish brain samples. **I** The activity of SOD in zebrafish intestinal samples. Data are expressed as means ± SD. *p < 0.05 indicate significant differences between exposure groups and the control group. #*p* < 0.05 indicate significant differences between vitamin D-high and vitamin D-low groups at the same PS-NPs concentration

Histopathological alterations of brain tissue after 21 days of exposure to 80-nm PS-NPs are shown in Fig. 2A. The blood–brain barrier basement membrane of the control group is relatively complete, but in 150 − , the basement membrane is damaged, and the integrity is visibly compromised. Importantly, compared with 150 − , the 150 + group has notably less damage, suggesting that vitamin D can alleviate some of this toxicity.

After exposure to PS-NPs for 21 days, the number of entries to the top, as well as active time and distance moved, decreased to different degrees in the PS-NPs-exposed group compared with controls, while the latency to entry the top half increased significantly in 150 − (Fig. 2B–E). The 15 + group exhibited increased number of entries to the top and active time compared with 15 − ; the 150 + group exhibited reduced latency to entry the top half of zebrafish compared with 150 − . The average velocity and average acceleration were unaffected by treatment (Figure S2A, B).

PS-NPs exposure decreased the neurotransmitters serotonin (5-HT), γ-aminobutyric acid (GABA), and dopamine (DA) significantly in the brain tissue (Fig. 4F–H). In addition, PS-NPs also led to an upward and downward trend in the content of cortisol and oxytocin (OT) (Figure S2C, D). Notably, the 150 + group exhibited significantly increased 5-HT content compared with 150 − . In addition, superoxide dismutase (SOD) activity in the brain tissue increased with PS-NPs, but adequate vitamin D slowed that rate of increase of SOD activity (Fig. 4I).

### Histopathological changes of the intestine and related parameters

**Fig. 3:**
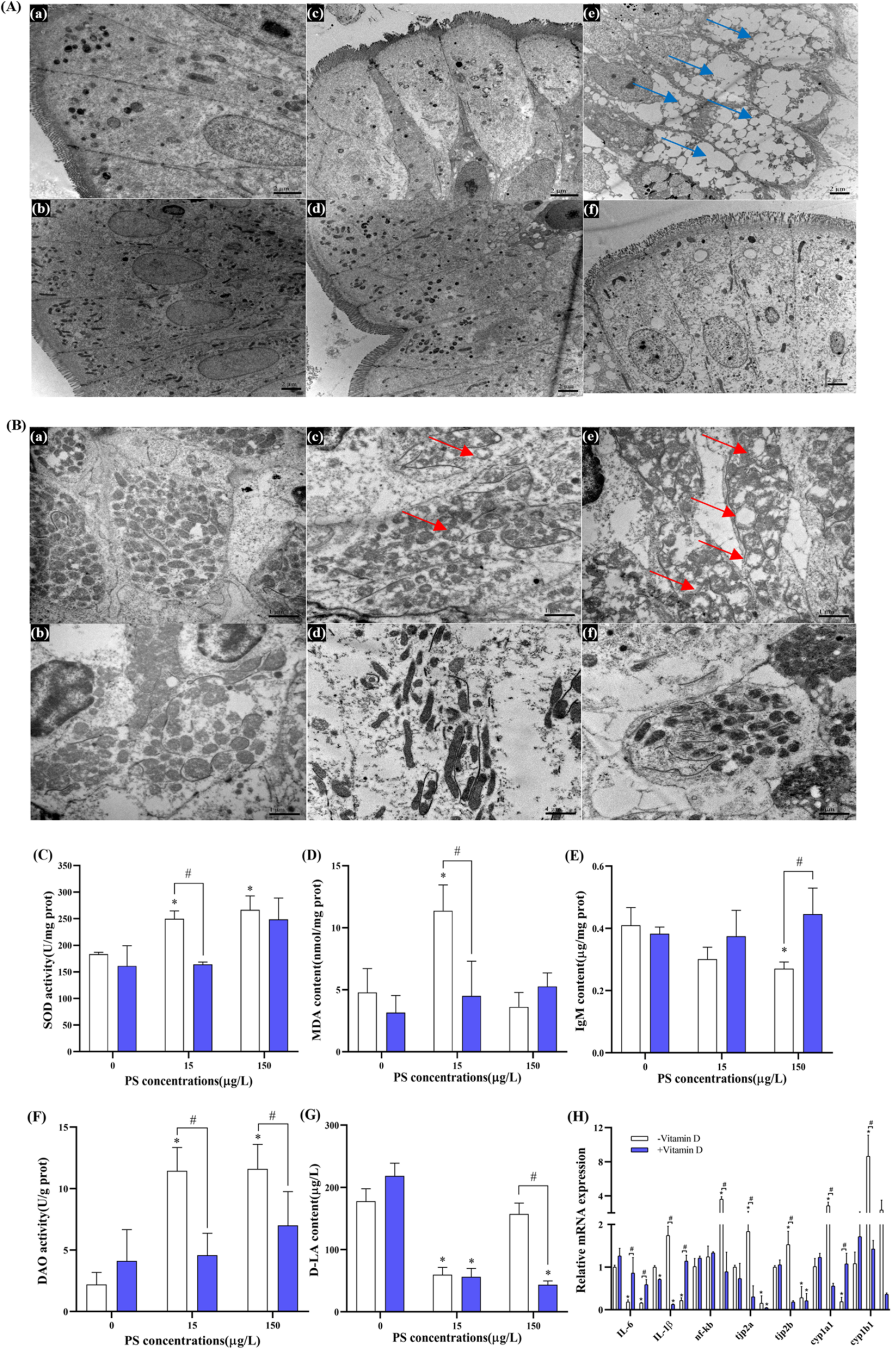
Representative histological changes of intestinal samples and changes of related parameters. **A** TEM of goblet cells (2 μm). **B** TEM of mitochondria (1 μm). a, b, c, d, e, and f represent the group of 0 − , 0 + , 15 − , 15 + , 150 − , and 150 + , respectively. The blue arrow represents the cytoplasmic matrix vacuolization, and the red arrow represents the mitochondrial vacuole. **C** The activity of SOD in zebrafish intestinal samples. **D** The content of MDA in zebrafish intestinal samples. **E** The content of IgM in zebrafish intestinal samples. **F** The activity of DAO in zebrafish intestinal samples. **G** The content of D-LA in zebrafish serum samples. **H** Expression of genes related to intestinal inflammation and permeability. Data are expressed as means ± SD. *p < 0.05 indicate significant differences between exposure groups and the control group; #*p* < 0.05 indicate significant differences between vitamin D-high and vitamin D-low groups at the same PS-NPs concentration

Histopathological alterations of the intestinal tissue are shown in Fig. 3A and B. The goblet cells and mitochondria in the 0 + group have no obvious alteration compared with controls. Goblet cells in the 15 − group showed slight cytoplasmic matrix vacuolization and a disorderly distribution of intestinal villi (Fig. 3A). Compared with 15 − , the intestinal villi of the 15 + group were arranged in a more orderly fashion. In the 150 − group, more serious vacuoles in cytoplasmic matrix were observed. Compared with 150 − , the 150 + group exhibited reduced vacuoles in cytoplasmic matrix, although there was still increased presence relative to the controls. The mitochondria also appeared to contain vacuoles after PS-NPs exposure, particularly in 150 − (Fig. 3B). Interestingly, these conditions were reduced in the vitamin D-high groups compared with the vitamin D-low groups.

The SOD activity of the 15 − and 150 − groups increased in a dose-dependent manner compared with controls (Fig. 3C), while malondialdehyde (MDA) content increased only in the 15 − group (Fig. 3D). Interestingly, the 15 + group exhibited a reversal of the increase in SOD activity and MDA content evident in 15 − exposure. Compared with controls, the high concentration of PS-NPs exposure led to a significant decrease of immunoglobulin M (IgM) content in the zebrafish intestinal tissues, while the vitamin D-high group restored levels to that of the unamended controls (Fig. 3E). In addition, the diamine oxidase (DAO) activity of the vitamin D-low group was significantly increased when exposed to different concentrations of PS-NPs, but there was no significant change in the vitamin D-high group (Fig. 3F). Compared with controls, exposure to the low-concentration PS-NPs resulted in a significant decrease of D-lactic acid (D-LA) in serum, although not in the 150 − group (Fig. 3G).

We then evaluated the effect of PS-NPs exposure on the expression of genes related to intestinal inflammation and permeability (Fig. 3H). Compared with controls, the expression of *IL-6* in 15 − and 150 − groups was decreased significantly, and the expression of the nuclear factor kappa-B (*nf-kb*) gene in 150 − group was increased significantly. Under the same conditions, higher vitamin D levels can minimize these trends. Interestingly, the low concentration PS-NPs led to the upregulation of *IL-1β* in the intestinal tissues, while the high concentration had the opposite effect. However, high vitamin D can reverse these changes under the same conditions. In addition, the expression of tight junction protein 2a (*tjp2a*), tight junction protein 2b (*tjp2b*), cytochrome 1a1 (*cyp1a1*), and cytochrome 1b1 (*cyp1b1*) increased significantly in 15 − , but importantly, expression was significantly decreased in 15 + .

### Virus metagenome sequencing

**Fig. 4:**
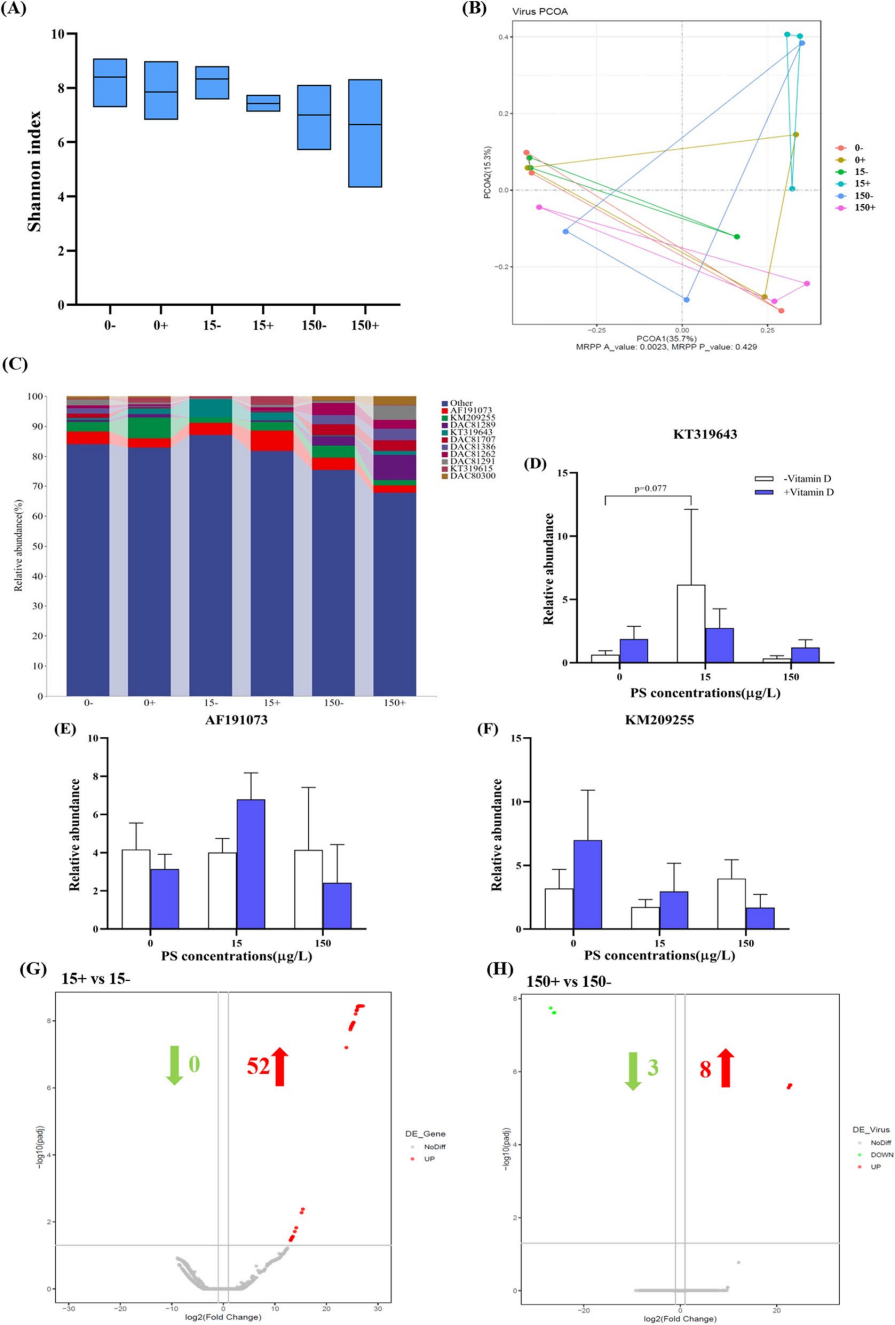
Virus metagenome sequencing of zebrafish intestinal content. **A** Alpha diversity (Shannon index) of zebrafish intestinal viruses. **B** The PCoA plot of zebrafish intestinal viruses. **C** Relative abundance of viruses at the species level (top 10). **D** The relative abundance of *KT319643* (UNVERIFIED: *Listeria* phage WIL-3). **E** The relative abundance of *AF191073* (stealth virus 1 clone 3B43). **F** The relative abundance of *KM209255* (*Dickeya* phage phiDP10.3 clone pD10). **G** Viruses with significant differences between the 15 + and 15 − group. **H** Viruses with significant differences between the 150 + and 150 − group. The green arrow represents downregulation, and the red arrow represents upregulation. DESeq2 was used for analysis, and viruses with FDR (false discovery rate) < 0.05 and fold change (FC) ≥ 2 were selected as significant differentially expressed viruses. Data are expressed as means ± SD. *p < 0.05 indicate significant differences between the exposure groups and the control group

After 21 days of exposure, the viruses in the intestines of zebrafish were characterized. The Shannon index and principal coordinate analysis (PCoA) show that different concentrations of PS-NPs not only reduced the diversity of the zebrafish gut virome but also disturbed the viruses community compared with the controls (Fig. 4A, B). Figure 4C shows the relative abundance of viruses in the control and exposure groups. The majority of the zebrafish gut virome can be regarded as “dark matter,” and 67–92% of the sequences are unidentified. Adenovirus is the main enriched virus, including *DAC81289*, *DAC81386*, *DAC81262*, *DAC81291*, *DAC80300*, and *DAC81707*, followed by two unverified *Listeria* bacteriophages, KT319643 and KT319615. Among these viruses, the abundance of *KT319643* (UNVERIFIED: *Listeria* phage WIL-3) in the 15 − group showed an obvious increasing trend compared with controls (Fig. 4D). The abundance of *AF191073* (stealth virus 1 clone 3B43) in the 15 + group (*t*-test, *p* = 0.037) and the abundance of *KM209255* (*Dickeya* phage phiDP10.3 clone pD10) in the 0 + group showed an obvious increasing trend compared with the controls (Fig. 4E, F). In the analysis of significantly differentially expressed viruses (DEVs), compared with 15 − , there were 0 DEVs downregulated and 52 DEVs upregulated in the 15 + group. Compared with 150 − , the 150 + group had 3 DEVs downregulated and 8 DEVs upregulated (Fig. 4G, H). The spliced contig sequence was then compared with the MiniKraken database, and the relative abundance of bacteria at the genus levels was obtained (Figure S3A). It was found that the abundance of *Exiguobacterium* increased significantly in 15 − , but the abundance of this group was then decreased in 15 + (Figure S3B).

### Correlation analysis

**Fig. 5:**
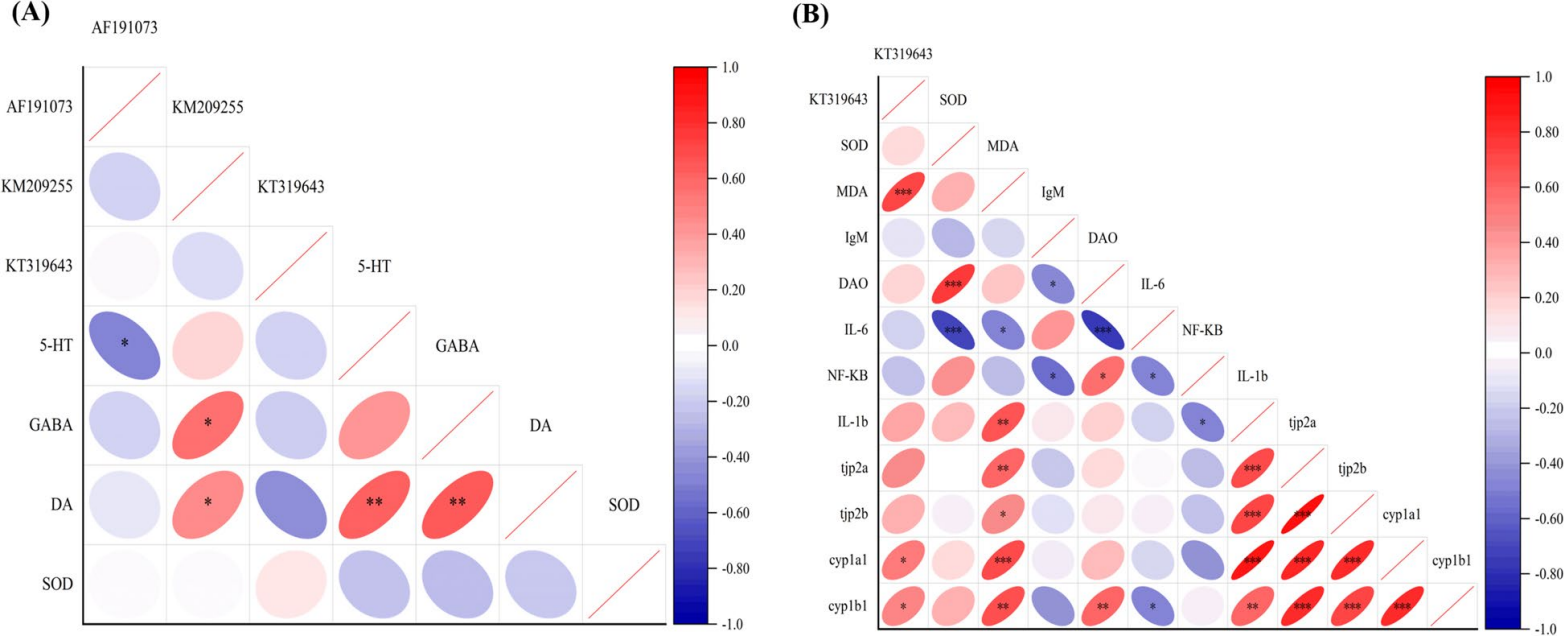
**A** Correlation analysis between viruses and brain biochemical indicators. **B** Correlation analysis between viruses and intestinal biochemical indicators. The smaller the oval area is, the larger the correlation coefficient is. Red represents a positive correlation, and blue represents a negative correlation. Pearson correlation analysis was used. *p ≤ 0.05, **p ≤ 0.01, ***p ≤ 0.001

In the correlation analysis between virus and brain-related indices, *AF1910731* was negatively correlated with the content of 5-HT in the zebrafish brain (*p* ≤ 0.05). In addition, *KM209255* was positively correlated with GABA and DA content in the zebrafish brain (*p* ≤ 0.05) (Fig. 5A). Therefore, it appears that *AF191073* and *KM209255* may be involved in the mediation of changes in neurotransmitters in the brain, which could then be a significant factor affecting the brain-gut axis interaction. In the correlation analysis between virus and intestinal-related indices, *KT3196431* was positively correlated with MDA content (*p* ≤ 0.001), *cyp1a1* (*p* ≤ 0.05), and *cyp1b1* (*p* ≤ 0.05) gene expression in the intestines (Fig. 5B). Thus, the abundance changes of *KT319643* may be important factors leading to oxidative stress and immunotoxicity in zebrafish intestines. It is clear that there are complex correlations among the related indices such as oxidative stress, inflammatory factors, and the virus community.

## Discussion

In vertebrates, cerebral capillaries form the specific selective blood–brain barrier (BBB) to ensure brain homeostasis and protect the CNS; it is known that damage to the BBB may lead to inflammation and neurodegeneration [35]. In the current study, we found that adequate vitamin D can inhibit the damage of BBB basement membrane caused by PS-NPs. In addition, adequate vitamin D can alleviate the effects of PS-NPs on the zebrafish neurobehavior, including increasing the times of entering the top, reducing the latency time of entering the upper half, and increasing the activity time and moving distance. Reduced upper explorations are typically considered as an indicator of heightened anxiety [36]. The results are consistent with Jin et al., who reported that PS-MPs exposure can lead to BBB damage in mice, correlating with cognitive and memory deficits [37].

To further investigate the neurobehavioral damage of PS-NPs and the mechanisms by which vitamin D can alleviate that toxicity, the expression levels of neurotransmitters in brain tissue were measured. Wei et al. showed that excessive cortisol levels may cause anxiety-like behavioral reactions, including longer latency and less time spent in the upper half of the aquarium [36]. Increased cortisol is considered to be a physiological sign of stress, demonstrating that exposure to PS-NPs in this study leads directly to anxiety-like behavior of the zebrafish [38]. In addition, DA and 5-HT are involved in the regulation of cognitive processes such as emotion, arousal, and memory in the CNS; GABA is the main inhibitory neurotransmitter that reduces the excitability of the CNS [39, 40]. In the PS-NPs treatment group, the content of 5-HT, GABA, and DA were all decreased to varying degrees, which further highlights the neurotoxicity of PS-NPs.

Vitamin D is pivotal in nerve development in many species, and deficiency can disrupt brain development and lead to lasting behavioral disorders [41–43]. For example, vitamin D deficiency led to the decrease of zebrafish vertical position and increases in panic reaction in a new tank diving experiment [41]. In addition, vitamin D deficiency has been shown to be related to some neurological diseases of humans, with cognitive decline being a common symptom [44–46]. Given this, vitamin D supplements may have use in the treatment and prevention of neurological diseases such as Alzheimer’s disease (AD). In fact, in the current study, adequate vitamin D significantly increased the level of 5-HT in zebrafish brain tissue compared with the vitamin D-low group, thereby reducing PS-NPs-induced neurotoxicity. In addition, vitamin D is the main regulator of calcium metabolism in many organisms, and studies have proved that calcium metabolism can regulate the release of neurotransmitters, so we infer that vitamin D may regulate the nervous system by affecting calcium metabolism in zebrafish, and further research is needed in the future [47].

It is generally believed that there is direct communication between the brain and intestine. For example, psychological stress has been linked to both changes in intestinal barrier function and food allergies [48]. We further analyzed the intestinal injury induced by PS-NPs to clarify the relationship between the zebrafish brain and intestine. Intestinal goblet cells play a central role in regulating intestinal health due to their providing a protective mucous layer covering the intestine, sensing of changes in the local environment, and forming intestinal immunity [49, 50]. Histopathological results showed that exposure to PS-NPs caused a large number of vacuoles in goblet cells and in mitochondria of the intestinal tissues, which may negatively affect the intestinal immune system and mitochondrial function. However, these phenomena were partially alleviated in the vitamin D-high group, which may be a function of less PS-NPs accumulation.

To clarify the mechanism of vitamin D-reduced toxicity in the intestine, several relevant biochemical parameters were determined. The increase of SOD activity in the zebrafish intestinal tract indicates oxidative stress, which is related to decreased mitochondrial function [51]. Adequate vitamin D can reduce mitochondrial vacuoles and protect mitochondrial function, thereby alleviating oxidative stress induced by PS-NPs. IgM is an important immune effector and plays a vital role in protection against various pathogens [52]. In the current study, the high concentration exposure PS-NPs decreased IgM content in the intestinal tissues, indicating a decrease of immune function that could then lead to damage of intestinal goblet cells from exogenous substances. Adequate vitamin D can increase the IgM level and improve immune function. In addition, it has been reported that changes in DAO activity are a biomarker of intestinal inflammation [53]. Here, DAO activity increased significantly with PS-NPs exposure, inducing intestinal inflammation, but importantly, high vitamin D returned DAO activity to baseline levels. These results indicate that adequate vitamin D can reduce the histological damage and inflammatory response induced by PS-NPs by improving zebrafish immune function.

To further probe the intestinal inflammation induced by PS-NPs and the regulation of vitamin D at the molecular level, the expression of genes related to intestinal inflammation and permeability was investigated. ILs are considered the driving force and key component of innate immunity [54]. *IL-1β* can be stimulated by stress response and works to recover and maintain the intestinal barrier; specifically, *IL-6* is a highly versatile inflammatory cytokine with pleiotropic and redundant activities [55, 56]. In the current study, PS-NPs resulted in the downregulation of *IL-6* and upregulation of *IL-1β*, indicating a strong stress response to exposure. The *nf-kb* controls the survival and proliferation of different proinflammatory mediators and cells [57]. Here, the expression level of *nf-kb* increased significantly in 150 − , suggesting the occurrence of inflammation. Tight junction protein is the intercellular junction complex in epithelial cells, and it is the key structure of barrier functional integrity [58]. The increase of the expression levels of *tjp2a* and *tjp2b* may alter the permeability of the intestinal barrier, thereby increasing the level of mucin that can harm intestinal microflora [12]. Previous reports indicate that overexpression of *cyp1a1* and *cyp1b1*, as downstream genes of the aromatic hydrocarbon receptor (AHR) pathway, contributes to intestinal inflammation and colorectal cancer risk, which is also indicative of PS-NPs-induced intestinal inflammation in zebrafish in this study [59, 60]. Surprisingly, high vitamin D levels can restore the expression of these genes to normal levels, confirming its importance on immunity.

There is growing evidence of vitamin D involvement in immune regulation and gut barrier function, as well as involvement in various regulatory pathways that prevent inflammation and immune-mediated tissue damage [61, 62]. For example, Triantos et al. showed that vitamin D positively affected intestinal tissues, resulting in improved barrier function and stronger innate immune response, and weakened adaptive immune response mediated by inflammatory T cells closely related to IBD [62]. Therefore, vitamin D is a better choice for regulating the adverse effects of intestinal damage caused by environmental pollutants.

Disturbance of intestinal microflora may cause local inflammation and dysregulate immune response, which is believed to cause neuroinflammation, as well as changes in brain function and behavior [63]. To analyze the role of the gut virome in the brain-gut axis relationship of zebrafish, the virus metagenome was sequenced. In this study, the decrease of serum D-LA content with PS-NPs indicates a metabolic disorder of intestinal flora, which may be related to changes of virus abundance [64]. The results of PCoA also confirmed the influence of PS-NPs on viral richness. Bacteriophages may act as regulators (lysogenic bacteriophages) or predators (lytic bacteriophages), resulting in changes in the composition of bacterial microbiome [65]. The DEVs between the vitamin D-low group and the vitamin D-high group include the following phages: *Bacillus *phage, *Serratia *phage, *Listeria *phage, *Lactococcus *phage, and *Salmonella* phage (Tables S1, S2). Studies have shown that *Bacillus*, *Serratia*, and *Lactococcus*, the hosts of the above bacteriophages, are related to the secretion of some neurotransmitters [66]. *Lactobacillus* and *Bifidobacterium* can secrete GABA, *Bacillus* and *Serratia* can secrete DA, and up to 80% of 5-HT in the gastrointestinal tract is produced by *Lactobacillus*, *Bifidobacterium*, *Bacteroides*, and *Lactococcus* [17, 67]. The current study suggests that the decrease of 5-HT, GABA, and DA content in zebrafish intestines might be due to the changes of the above virus groups in the intestinal tract, or the change of host bacteria abundance impacted by those viruses, which subsequently affected nerve transmission and behavior [20]. Furthermore, among these DEVs, the *Lactococcus* virus is known to be related to better performance of the central executive system [26]. Thus, changes in the abundance of these bacteriophages or viruses may be an important element that mediates vitamin D regulation of the brain-gut axis relationship. Importantly, this is the first study that links zebrafish gut virome to neurotransmitters, opening a new line of toxicology research related to zebrafish enteroviruses.

Next, we investigated changes in bacterial abundance. *Exiguobacterium* belongs to gram-positive bacteria and can biotransform styrene monomers into 2-hydroxypenta-2,4-dienoate and acrylic acid [68]. This may also be why some indicators are more prominent at the environmentally relevant concentration of PS-NPs; that is, *Exiguobacterium* degrades and metabolizes polystyrene into other more toxic substances such as acrylic acid. In addition, the reason for this nonmonotonic change may be that there is an adaptive mechanism at the high concentration of PS-NPs [20, 69]. This suggests that PS-NPs at environmentally relevant concentrations have higher than expected toxicity; further research is needed to explore this important topic.

It is clear from the above discussion that *AF191073* and *KT319643* may be key viruses that mediate the vitamin D reduction of PS-NPs-induced neurotoxicity, intestinal oxidation, and metabolic toxicity. There are limited reports about *AF191073* (stealth virus), which is a double-stranded DNA virus belonging to Herpesviridae [70]. Sequence reads of the stealth virus have been found in the serum of patients with acute hepatitis; however, its pathophysiological function is still unclear. Studies have noted that stealth virus is also associated with some acute encephalopathy. Children with vacuole stealth virus encephalopathy are initially characterized by repeated behavior problems, with obvious noninflammatory vacuoles evident on brain biopsy [71]. *Listeria monocytogenes*, the host of *Listeria* phage (*KT319643*), can cause listeriosis, which can manifest as septicemia, meningitis, or meningoencephalitis [72]. Correlation analysis provides an understanding of intestinal viruses and key biochemical indicators leading to zebrafish behavior changes and immune damage and also provides insight into the role of viruses in brain-gut axis interaction. These results increase mechanistic understanding on the effect of vitamin D-induced alleviation of the toxicity of PS-NPs on the brain-gut axis of zebrafish.

## Conclusions

This study presents the first analysis of the zebrafish gut virome. There is clear evidence that exposure of zebrafish to PS-NPs can lead to anxiety-like behavior and intestinal immune damage. Importantly, vitamin D can relieve brain-gut axis dysregulation by mediating changes of gut virome. Specifically, vitamin D can alleviate neurotoxicity and immunotoxicity induced by PS-NPs exposure by directionally altering the gut virome. Our research provides insight into how PS-NPs can disrupt the brain-gut-virome axis. The current findings demonstrate that more research is needed to undestand the risk of PS-NPs at environmentally relevant concentrations. In addition, the role of vitamin D in moderating the damage of PS-NPs to the brain-gut axis of zebrafish is a significant finding with broad implications.

## Methods

### Chemicals and materials

A dispersed PS-NPs solution (80 nm in diameter) without sodium azide was purchased from Tianjin BaseLine ChromTech Research Centre (Tianjin, China). We use spherical PS-NPs to simplify the exposure and better analyze the influence of PS-NPs on the brain-gut axis of zebrafish. The solution was diluted with deionized water to obtain 10 mg/L PS-NPs stock solution. To prevent PS-NPs from agglomerating, the solution was sonicated for 15 min before every usage at a frequency of 100 HZ.

Two fish diets were purchased from Trophic Animal Feed High-Tech Co. Ltd. (Jiangsu, China): a vitamin D-low diet (280 IU/kg) and a vitamin D-high diet (2800 IU/kg) [73]. In fact, the normal diet of zebrafish also contains VD. The vitamin D-low diet is relative to vitamin D-high diet.

### Zebrafish maintenance and experimental design

A 2-month-old healthy zebrafish were purchased from the Beijing aquarium and maintained under light/dark = 14/10 h conditions at 26 ± 1 °C in water. According to previously published work, the estimated environmental concentrations of 50–100 nm NPs in aquatic environments range from 1 pg/L to 15 μg/L [14]. In this study, we conducted experiments at environmentally realistic concentrations of PS-NPs at 15 μg/L, as well as a level that was tenfold higher 150 μg/L to understand the dose–response relationship. After 7 days of acclimation, the zebrafish (each group had about 60 fish) were exposed to 0, 15, and 150 μg/L PS-NPs in 20-L glass tanks and were concomitantly fed with zebrafish model diet (280 IU/kg or 2800 IU/kg) three times per day, thus yielding six treatments: 0 − , 15 − , and 150 − indicating feeding vitamin D-low diet and 0 + , 15 + , and 150 + indicating feeding vitamin D-high diet. The exposure solution was replaced every 2 days, which can ensure the water quality and prevent PS-NPs from gathering in the water. After 21 days of exposure, zebrafish was anesthetized with MS-222 (100 mg/L tricaine and 300 mg/L NaHCO_3_) and put on ice for keeping biological activity of tissues. After excision of caudal fin, zebrafish plasma was collected by capillary tubes. Zebrafish were dissected on ice, extracted the brain and intestine, and stored at − 80 °C for further analysis.

### Determination of growth parameters

Zebrafish in each group were weighed and measured to evaluate the influence of different concentrations of PS-NPs on growth as follows: *K* = weight (g) × 100/length^3^ (cm)), *BSI* = brain weight × 100/total weight, and *ISI* = intestine × 100/total weight of zebrafish (*n* = 8 replicates). To analyze FI, 10 zebrafish were randomly selected from each group and placed in another aquarium while maintaining the same exposure conditions. A specific amount of food was added to each aquarium, and the food is fixed in the aquarium through the red worm feeding cup. After 24 h, the remaining food was collected and weighed, and the process was repeated three times (*n* = 3 replicates). FI was calculated as *FI* = *W*_i_-(*W*_f_ × F), where *W*_i_ = initial dry food weight, *W*_f_ = remaining dry food weight, and *F* = correction factor (the effect of water dissolution on food particles in the absence of fish).

### Histopathological analysis

The intestine and brain samples of zebrafish were added into a microscope fixative solution for subsequent observation by TEM (Hitachi Limited, Japan). The protocol includes conventional sample preparation, ultrathin slicing, and TEM observation [9]. Detailed information can be found in Text S1. We also observed NPs using TEM through its characteristics, such as particle size (80 nm), sphericity, and opacity.

### Biochemical measurement

The enzymatic activities of SOD and DAO and the content of MDA in brain or intestine of zebrafish were determined using manufacturer kits (Nanjing Jiancheng Biological Co. Ltd., China) (*n* = 3 replicates). The contents of IgM, GABA, 5-HT, DA, cortisol, and OT in the brain or intestine of zebrafish were determined by an ELISA kit (Gene-Lab Inc., Beijing, China). The contents of D-LA in serum of zebrafish were also determined by an ELISA kit (Gene Lab Inc., Beijing, China). ELISA analysis involves antigens, or antibodies in the sample are adsorbed on the surface of solid carrier, followed by incubation with enzyme-labeled (coupled) antibody or antigen, the addition of a chromogenic agent to develop color. Then one can measure the color difference between the sample and standard spectrophotometrically and construct an enzyme activity curve to calculate the concentration of the test object. All parameters were normalized against protein content. The protein concentration was measured using the BCA assay kit (Nanjing Jiancheng Biological Co. Ltd., China). Manufacturer protocols were followed in all assays. Detailed information can be found in Text S2.

### Quantitative real-time polymerase chain reaction assays (qRT-PCR)

The intestines from three fish were pooled together as a single replicate (*n* = 3 replicates), and the total RNA was extracted with a TRIzol solution. The RNA concentration of each sample was measured using NanoDrop 2000 spectrophotometer (Thermo Fisher Scientific Inc., USA). The first-strand cDNA synthesis was performed according to the manufacturer’s instructions (Tiangen Inc., Beijing, China). Two micrograms of total RNA samples was synthesized in the complementary DNA in 20-μL reaction system. Targeted genes were investigated by qRT-PCR assays. The process of qRT-PCR was operated in a 20-μL reaction and conducted with an ABI 7500 system (Advanced Biosystems, Foster City, CA, USA). The qRT-PCR conditions were set at 95 °C for 15 min, and 40 cycles of 10 s at 95 °C, 20 s at 60 °C, and melting curve analysis of 32 s at 72 °C. All primers used are described in Table S3. The housekeeping gene was* β*-actin. The relative expression between the control and treatments was compared using the 2^−ΔΔCt^ method.

### Novel tank diving test

The principle of the novel tank diving test is that when zebrafish are introduced into a new environment, individals will dive to the bottom first and then move up slowly over time. The diving device is a 1.5-L trapezoidal tank that is filled with water and divided into two sections (top and bottom) by the line marking the outer wall. Before the exposure, zebrafish were put into a glass beaker and adapted to the new environment for 1 h. The fish were then introduced into the novel tank within 3 s. The behavior of each fish in the novel tank was recorded for 6 min by the Video-Track system (UI-3240CP-C-GL, IDS Imaging Development System GmbH, Obersulm, Germany), and different behavior endpoints were recorded, including the latency to reach the top half of the tank (s) and the number of entries to the top (*n* = 5 replicates). In addition, the recorded video was further analyzed by the uEye Cockpit software (Loligo Systems, USA), calculating the average speed (mm/s), average acceleration (mm/s^2^), active time (s), and total distance traveled (mm).

### Gut virome analysis

Ten zebrafish intestinal contents in each group were collected in a sterile tube (*n* = 3 replicates). These samples were stored in dry ice and sent to Tiangen Inc. for virus metagenome sequencing. Classification and annotation of the contigs were obtained by splicing and detection of species distribution. Alpha diversity is determined by calculating the Shannon index. PCoA was used to reveal the differences in virus communities among different exposure groups. The classified database used is NCBI RefSeq viral genomes and viral nucleic acid sequences in GenBank. Candidate viruses were obtained through comprehensive screening of BlastN and BlastX comparison algorithms. In addition, the spliced contig sequence was compared with the MiniKraken database by Kraken software, and the relative abundance of bacteria was obtained. Detailed information can be found in Text S3.

### Statistical analysis

Statistical analysis was carried out the IBM SPSS Statistics 26.0. Normality and homogeneity of variance were evaluated using Skewness test and Levene’s test, respectively. Statistical differences were determined by one-way analysis of variance (ANOVA) with Dunnett multiple-comparison test. Data were graphed using GraphPad Prism software. *p < 0.05 indicates significant differences between exposed groups and the control group; #*p* < 0.05 indicates significant differences between vitamin D-high and vitamin D-low groups at the same PS-NPs concentration. Data are expressed as mean ± SD. Heatmap was graphed, and Pearson correlation analysis was used by Origin. *p* < 0.05 are considered to be of statistical significance.

### Supplementary Information

**Additional file 1: Text S1. **TEM analysis. **Text S2.** Determination of biochemical parameters. **Text S3.** Gut Virome Analysis. **Figure S1.** Accumulation of PS-NPs in the brain tissues of zebrafish and changes of related growth parameters (0.2 μm). (A) TEM observation of PS-NPs in brain tissue, (a), (b), (c), (d), (e), and (f) represent the group of 0-, 0+, 15-, 15+, 150-, and 150+, respectively, the blue arrow points to the NPs; (B) The number of NPs in brain tissue (*n*=3 replicates); (C) BSI (%). Data are expressed as means±SD. *p<0.05 indicate significant differences between the exposure groups and the control group. **Figure S2.** (A) Average velocity (mm/s); (B) Average acceleration (mm/s2); (C) and (D) represents the content of cortisol and OT in zebrafish brain samples. Data are expressed as means±SD. *p<0.05 indicate significant differences between the exposure groups and the control group. **Figure S3.** (A) Relative abundance of bacteria at the genus level (top 10) (*n*=3 replicates); (B) The relative abundance of Exiguobacterium. Data are expressed as means±SD. *p<0.05 indicate significant differences between exposure groups and the control group; #*p*<0.05 indicate significant differences between vitamin D-high and vitamin D-low groups at the same PS-NPs concentration. **Table S1.** Differentially expressed virus in 15+ vs 15- comparison. **Table S2.** Differentially expressed virus in 150+ vs 150- comparison. **Table S3.** Primer information used in qRT-PCR. All sequences are shown 5’-3’.

**Additional file 2.**

## Data Availability

16S rRNA gene sequencing raw data are deposited into the GenBank SRA database under the BioProject PRJNA974094 (https://dataview.ncbi.nlm.nih.gov/object/PRJNA974094?reviewer=9dvo9h0hgg82pjrfll79jovava). Results of the pathological alterations, biochemical assay, and correlated analysis are available in the supplementary materials.
