# Supplementary material for: Vitamin D modulation of brain-gut-virome disorder caused by polystyrene nanoplastics exposure in zebrafish (Danio rerio)
Source: Microbiome. 2023 Nov 27;11:266. doi: 10.1186/s40168-023-01680-1 (PMC10680193; doi:10.1186/s40168-023-01680-1)
Supplement: Supplementary file 2 — Additional file 1: Text S1. TEM analysis. Text S2. Determination of biochemical parameters. Text S3. Gut Virome Analysis. Figure S1. Accumulation of PS-NPs in the brain tissues of zebrafish and changes of related growth parameters (0.2 μm). (A) TEM observation of PS-NPs in brain tissue, (a), (b), (c), (d), (e), and (f) represent the group of 0-, 0+, 15-, 15+, 150-, and 150+, respectively, the blue arrow points to the NPs; (B) The number of NPs in brain tissue (n=3 replicates); (C) BSI (%). Data are expressed as means±SD. *p<0.05 indicate significant differences between the exposure groups and the control group. Figure S2. (A) Average velocity (mm/s); (B) Average acceleration (mm/s2); (C) and (D) represents the content of cortisol and OT in zebrafish brain samples. Data are expressed as means±SD. *p<0.05 indicate significant differences between the exposure groups and the control group. Figure S3. (A) Relative abundance of bacteria at the genus level (top 10) (n=3 replicates); (B) The relative abundance of Exiguobacterium. Data are expressed as means±SD. *p<0.05 indicate significant differences between exposure groups and the control group; #p<0.05 indicate significant differences between vitamin D-high and vitamin D-low groups at the same PS-NPs concentration. Table S1. Differentially expressed virus in 15+ vs 15- comparison. Table S2. Differentially expressed virus in 150+ vs 150- comparison. Table S3. Primer information used in qRT-PCR. All sequences are shown 5’-3’. [file 40168_2023_1680_MOESM1_ESM.docx]

**Vitamin D modulation of brain-gut-virome disorder caused by polystyrene nanoplastics exposure in zebrafish (*Danio rerio*)**

Miaomiao Teng^1#^, Yunxia Li^1#^, Xiaoli Zhao^1*^, Jason C. White^2^, Lihui Zhao^1^, Jiaqi Sun^3^, Wentao Zhu^4^, Wentian Zhao^4^, Fengchang Wu^1*^

1. State Key Laboratory of Environmental Criteria and Risk Assessment, Chinese Research Academy of Environmental Sciences, Beijing 100012, China
2. The Connecticut Agricultural Experiment Station, New Haven Connecticut 06511, U.S.A.
3. School of Energy and Environmental Engineering, University of Science and Technology Beijing, Beijing 100083, China
4. Innovation Center of Pesticide Research, Department of Applied Chemistry, College of Science, China Agricultural University, Beijing 100193, China

*Corresponding Authors:

Fengchang Wu

E-mail: [wufengchang@vip.skleg.cn](mailto:wufengchang@vip.skleg.cn)

Xiaoli Zhao

E-mail: [zhaoxiaoli_zxl@126.com](mailto:zhaoxiaoli_zxl@126.com)

State Key Laboratory of Environmental Criteria and Risk Assessment, Chinese Research Academy of Environmental Sciences, Beijing 100012, China

Address: Anwai Dayang Fang 8#, Chaoyang District, Beijing, China

**Text S1**

**Transmission electron microscopy (TEM) analysis.** The protocol includes conventional sample preparation by TEM, ultrathin slice, and observation by TEM. (1) conventional sample preparation of TEM: washing the fixing solution completely with buffer solution; Fix with 1% osmic acid for 2h; Gradient dehydration with 30, 50, 70, 80, 90, 95, 100% alcohol; Replace alcohol with anhydrous acetone; Soak overnight in a 35℃ incubator with the mixture of embedding agent and acetone; After marking, embedding the sample into an embedding plate; The embedded sample is placed in a temperature box, and the temperature is raised step by step for polymerization. (2) Ultra-thin section: the tissue is trimmed into a four-sided cone with a smooth top surface and exposed target tissue; Making glass cutter with a knife-making machine; Leica UC6 ultrathin slicer slices; Uranium acetate and lead citrate double staining. (3) Observation with transmission electron microscope: observe with H-7500 TEM of Hitachi, Japan, working voltage 80 kV, multiple ranges 700-200,000 times. We randomly select tissue slice areas under the same magnification and field of view, and counted the number of NPs in each field of view (n=3). Because there is no reliable quantitative method to measure the histological damage caused by PS-NPs in this study, a qualitative method is used and several typical pictures are selected for display.

**Text S2**

**Determination of biochemical parameters.** Brain and intestinal samples were weighed and homogenized with a tissue homogenizer using physiological saline. The homogenate was centrifuged at 2500 rcf for 15 min at 4 ℃ to obtain supernatant, which was used to measure the activities of superoxide dismutase (SOD), malondialdehyde (MDA), and diamine oxidase (DAO) according to the manufacturer protocols (Nanjing Jiancheng Bioengineering Research Institute, China) and the contents of immunoglobulin M (IgM), D-lactic acid (D-LA), γ-aminobutyric acid (GABA), serotonin (5-HT), dopamine (DA), cortisol, and oxytocin (OT) according to the manufacturer protocols (Gene-lab Inc., Beijing, China). ELISA analysis involves antigens or antibodies in the sample are adsorbed on the surface of solid carrier, followed by incubation with enzyme-labeled (coupled) antibody or antigen, the addition of a chromogenic agent to develop color. Then one can measure the color difference between the sample and standard spectrophotometrically, and construct an enzyme activity curve to calculate the concentration of the test object. The supernatant was diluted 20 times for protein analysis. The protein content of brain and intestinal samples were determined following the manufacturer’s instructions (BCA assay kit, Nanjing Jiancheng Biological Co. Ltd., China). Standard curves of protein were obtained to measure the amount of sample (R^2^>0.99). All samples were conducted in triplicate and quantified by protein.

**Text S3**

**Gut Virome Analysis.** The experimental process of virus metagenome sequencing includes: (1) virus metagenome sample detection. The detection of DNA samples mainly includes two methods: Analysis of DNA degradation degree and whether there is pollution by Agarose Gel Electrophoresis; Qubit accurately quantifies DNA concentration. (2) library construction. The qualified DNA samples were randomly broken into fragments with a length of about 350 bp by Covaris ultrasonic crusher, and the whole library was prepared by terminal repair, adding A-tail, adding sequencing linker, purification, PCR amplification and other steps. (3) library inspection. After the construction of the library, the library was initially quantified by using Qubit2.0, and the library was diluted to 2 ng/μL. Then, the insert size of the library was detected by using Agilent 2100. After the insert size met expectations, the effective concentration of the library was accurately quantified by using Q-PCR method (the effective concentration of the library was greater than 3 nM) to ensure the quality of the library. (4) computer sequencing. After the library inspection is qualified, the different libraries are pooling according to the effective concentration and the demand of the target off-line data, and then sequenced. After that, the sequencing results were analyzed by virus metagenome, including quality control, de-host reference genome, megaHit assembly, and contig classification annotation. The database used for classification includes nucleic acid sequence and protein sequence in NCBI Refseq Viral Genomes and virus sequence in GenBank. Finally, candidate viruses are summarized and further analyzed. The comparison algorithm adopts BlastN and BlastX and the candidate virus types are the comprehensive screening results of BlastN and BlastX. We compare the number of reads of candidate viruses, get the count number of candidate viruses in the sample, and count the relative abundance of the top 10 candidate viruses in each sample. The input data of differential expression virus is read count data obtained from virus abundance analysis. We use DESeq2 for the analysis of biological duplication samples. Finally, viruses with FDR (false discovery rate) less than 0.05 and fold change (FC) greater than or equal to 2 are listed as differentially expressed viruses. Kraken is an ultra-fast and highly accurate software for sequence taxonomy allocation. Based on the precise alignment of k-mers, the software achieves the classification accuracy equivalent to that of BLAST program. The software provides a reference database of bacteria, archaea and virus genomes, and we use Kraken software to compare the spliced Contig sequence with the MiniKraken database, and got the relative abundance of bacteria at genus levels.


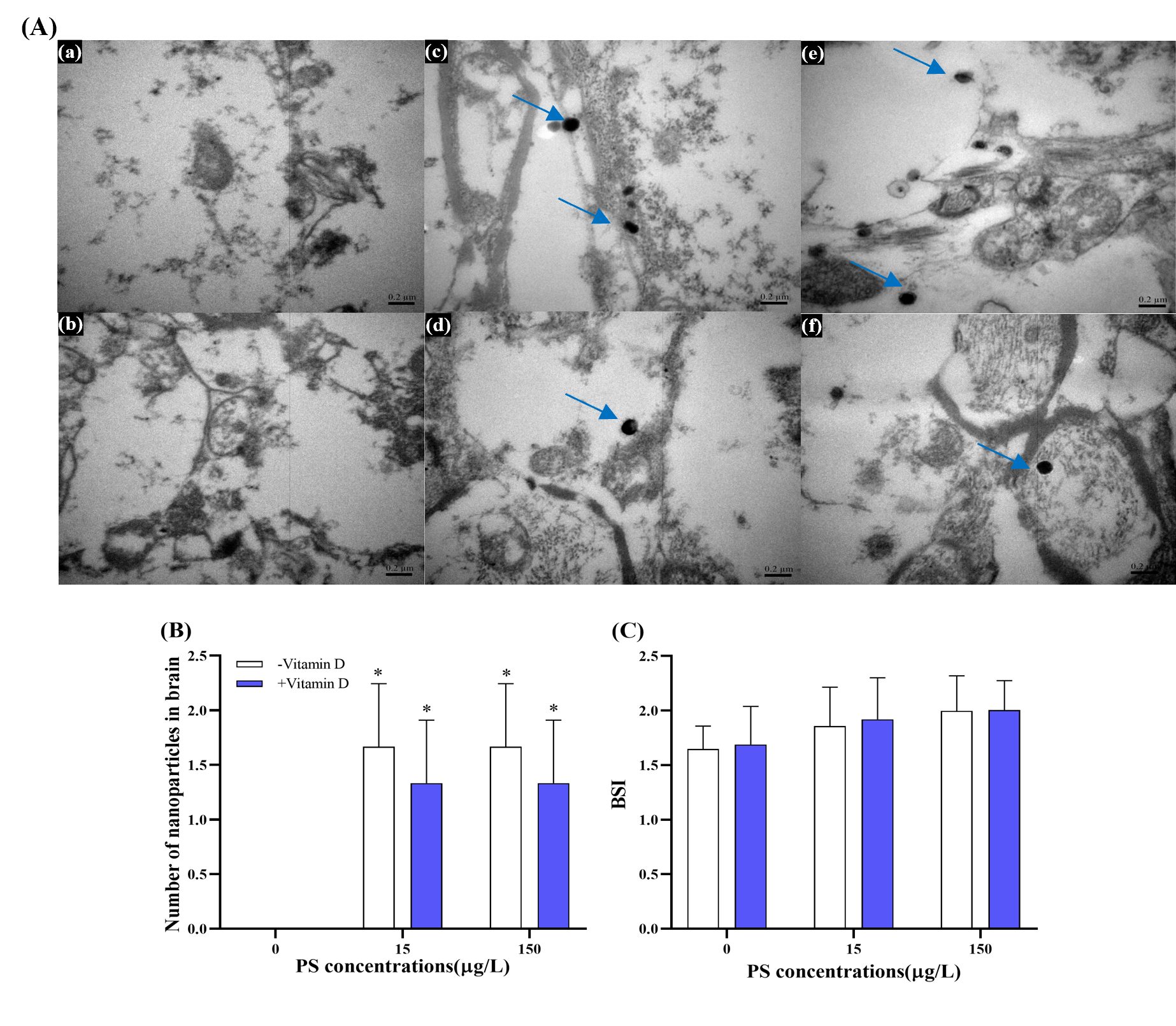


Figure S1. Accumulation of PS-NPs in the brain tissues of zebrafish and changes of related growth parameters (0.2 μm). (A) TEM observation of PS-NPs in brain tissue, (a), (b), (c), (d), (e), and (f) represent the group of 0-, 0+, 15-, 15+, 150-, and 150+, respectively, the blue arrow points to the NPs; (B) The number of NPs in brain tissue (n=3 replicates); (C) BSI (%). Data are expressed as means±SD. **p*<0.05 indicate significant differences between the exposure groups and the control group.


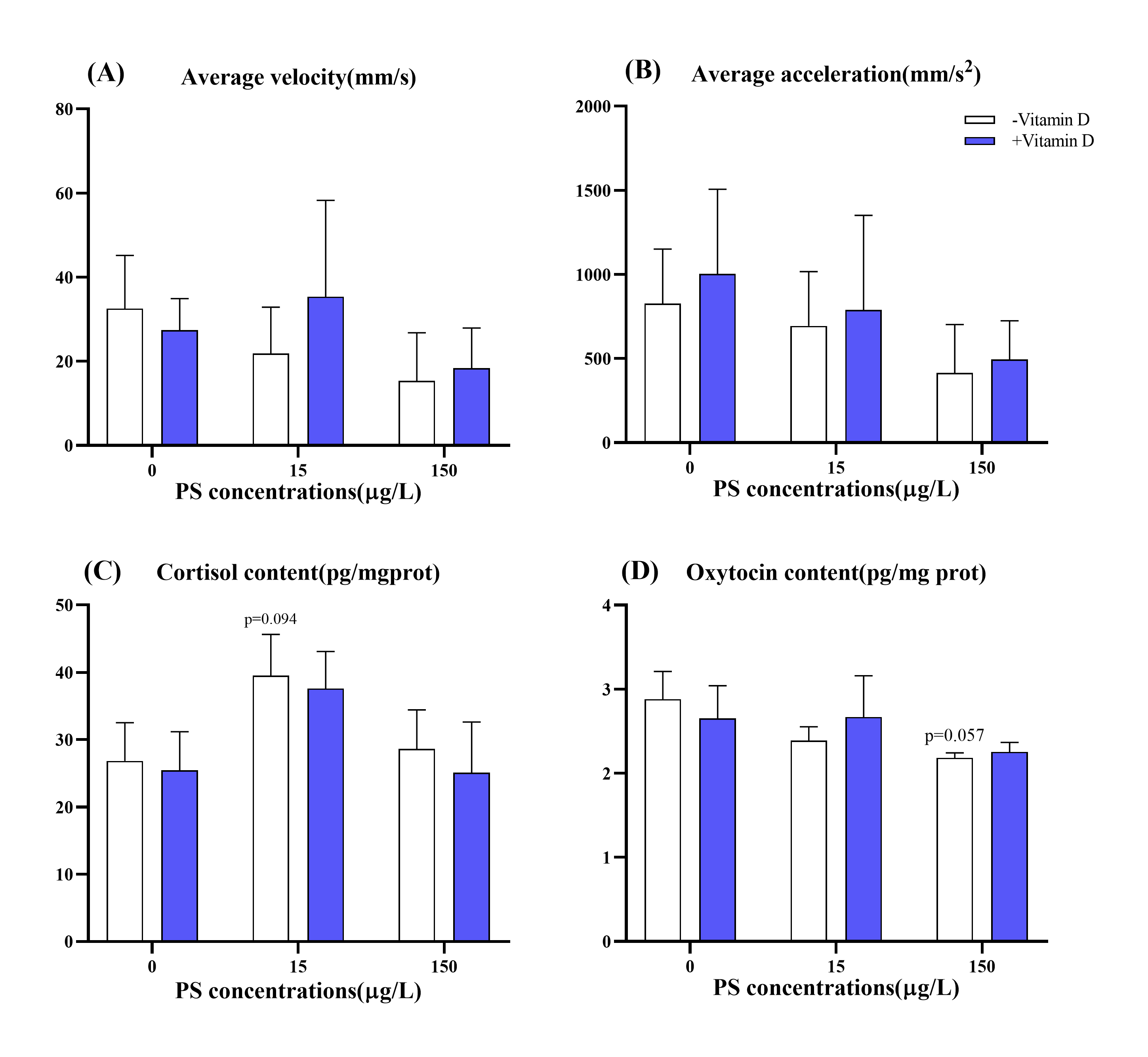


Figure S2. (A) Average velocity (mm/s); (B) Average acceleration (mm/s^2^); (C) and (D) represents the content of cortisol and oxytocin (OT) in zebrafish brain samples. Data are expressed as means±SD. **p*<0.05 indicate significant differences between the exposure groups and the control group.


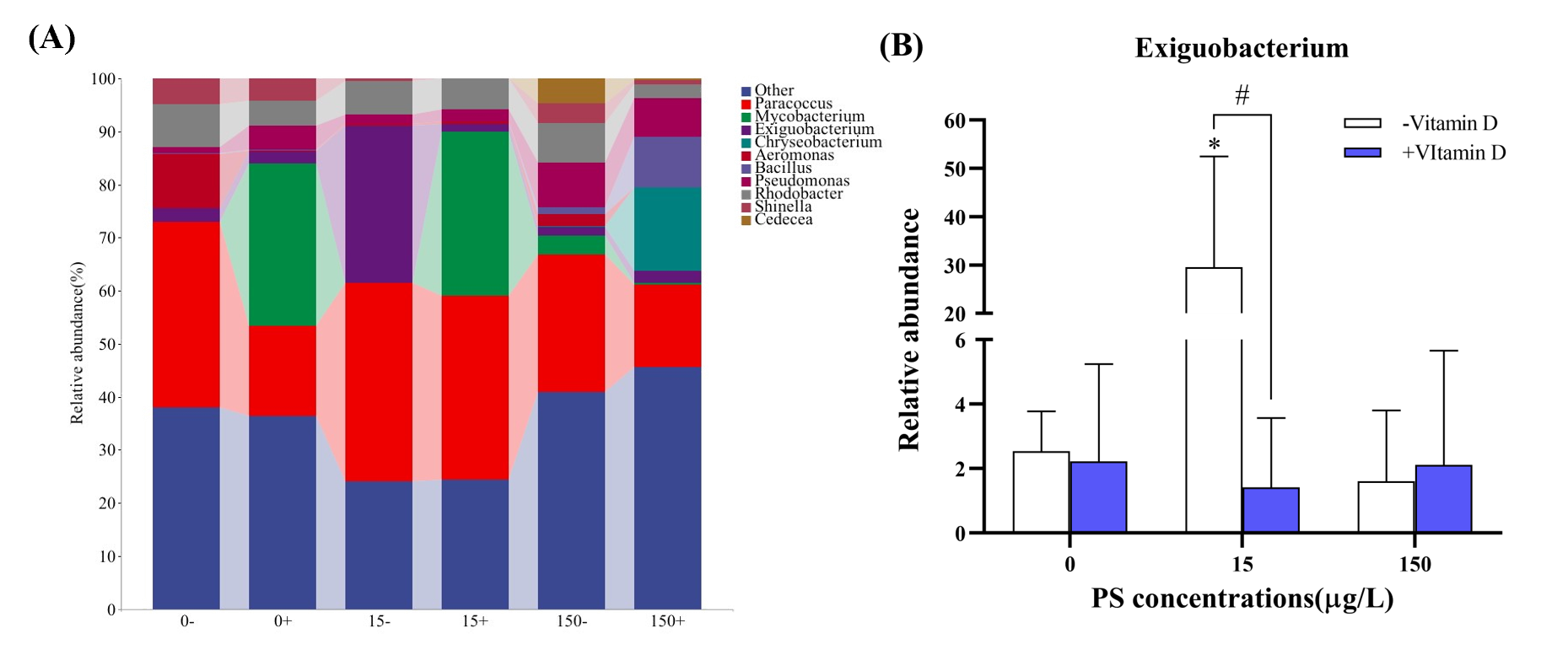
Figure S3. (A) Relative abundance of bacteria at the genus level (top 10); (B) The relative abundance of *Exiguobacterium*. Data are expressed as means±SD. **p*<0.05 indicate significant differences between exposure groups and the control group; #*p*<0.05 indicate significant differences between vitamin D-high and vitamin D-low groups at the same PS-NPs concentration.

Table S1. Differentially expressed virus in 15+ vs 15- comparison

| **VirusID** | **description** | **15+1** | **15+2** | **15+3** | **15-1** | **15-2** | **15-3** | **Pvalue** | **Padj** | **trend** |
| --- | --- | --- | --- | --- | --- | --- | --- | --- | --- | --- |
| MT154980 | HIV-1 isolate HK_JIDLNBL_S071 from Switzerland nonfunctional gag protein (gag) gene, complete sequence; and nonfunctional pol protein (pol) gene, partial sequence | 0 | 0 | 801.0332 | 0 | 0 | 0 | 4.08E-12 | 3.59E-09 | UP |
| MF468140 | Macacine betaherpesvirus 3 clone BAC RhCMV_68-1_85B/ESAT6, complete genome | 0 | 0 | 638.5047 | 0 | 0 | 0 | 7.28E-12 | 3.59E-09 | UP |
| QHJ82361 | hypothetical protein [Siphoviridae sp.] | 0 | 0 | 580.4588 | 0 | 0 | 0 | 9.32E-12 | 3.59E-09 | UP |
| QGJ84469 | transposase/integrase [Lactococcus phage proPhi1] | 0 | 0 | 545.6313 | 0 | 0 | 0 | 1.09E-11 | 3.59E-09 | UP |
| VUD36879 | putative lactone hydrolase, alpha/beta fold family [Escherichia virus Lambda_2G7b] | 0 | 0 | 534.0221 | 0 | 0 | 0 | 1.16E-11 | 3.59E-09 | UP |
| KT630647 | Salmonella phage SEN8, complete genome | 0 | 0 | 510.8038 | 0 | 0 | 0 | 1.29E-11 | 3.59E-09 | UP |
| YP_007877493 | hypothetical protein VPAG_00068 [Vibrio phage douglas 12A4] | 0 | 0 | 522.413 | 0 | 0 | 0 | 1.30E-11 | 3.59E-09 | UP |
| CAB4122922 | CbpA DnaJ-class molecular chaperone [uncultured Caudovirales phage] | 0 | 0 | 475.9762 | 0 | 0 | 0 | 1.54E-11 | 3.59E-09 | UP |
| YP_009196406 | putative RNA ligase 1 and tail fiber attachment catalyst [Cronobacter phage S13] | 199.5619 | 0 | 0 | 0 | 0 | 0 | 1.80E-11 | 3.59E-09 | UP |
| YP_009196542 | hypothetical protein S13_158 [Cronobacter phage S13] | 199.5619 | 0 | 0 | 0 | 0 | 0 | 1.80E-11 | 3.59E-09 | UP |
| YP_009847075 | ribonucleotide reductase A subunit [Aeromonas phage 4_4572] | 199.5619 | 0 | 0 | 0 | 0 | 0 | 1.80E-11 | 3.59E-09 | UP |
| YP_007877952 | glucose 6-phosphate dehydrogenase [Cyanophage Syn30] | 0 | 433.5399 | 0 | 0 | 0 | 0 | 1.93E-11 | 3.59E-09 | UP |
| YP_009805925 | DNA polymerase I [Salmonella phage S147] | 0 | 406.0135 | 0 | 0 | 0 | 0 | 2.28E-11 | 3.77E-09 | UP |
| MN184887 | Erwinia phage pEp_SNUABM_01, complete genome | 0 | 0 | 487.5854 | 0 | 0 | 0 | 2.54E-11 | 3.77E-09 | UP |
| QKU35457 | arylsulfatase [Tupanvirus soda lake] | 0 | 0 | 487.5854 | 0 | 0 | 0 | 2.54E-11 | 3.77E-09 | UP |
| YP_009882636 | tail sheath protein [Klebsiella phage ST512-KPC3phi13.2] | 0 | 350.9609 | 0 | 0 | 0 | 0 | 3.26E-11 | 4.54E-09 | UP |
| YP_009483662 | FAD linked glycolate oxidase [Pandoravirus quercus] | 0 | 337.1977 | 0 | 0 | 0 | 0 | 3.66E-11 | 4.80E-09 | UP |
| CAB5220421 | CobT, cobaltochelatase, CobT subunit [uncultured Caudovirales phage] | 0 | 323.4345 | 0 | 0 | 0 | 0 | 4.00E-11 | 4.95E-09 | UP |
| DAC81355 | TPA_asm: integrase [Nephila orb-weaver spider adintovirus] | 0 | 289.0266 | 0 | 0 | 0 | 0 | 5.29E-11 | 6.21E-09 | UP |
| MF975720 | Pseudomonas phage VW-6S, complete genome | 216.4739 | 0 | 0 | 0 | 0 | 0 | 1.06E-10 | 1.11E-08 | UP |
| MG250483 | Aeromonas phage Ah1, complete genome | 213.0915 | 0 | 0 | 0 | 0 | 0 | 1.11E-10 | 1.11E-08 | UP |
| CAB4168797 | HepA Superfamily II DNA/RNA helicases, SNF2 family [uncultured Caudovirales phage] | 213.0915 | 0 | 0 | 0 | 0 | 0 | 1.11E-10 | 1.11E-08 | UP |
| YP_009289241 | transposase [Pseudomonas phage MD8] | 209.7091 | 0 | 0 | 0 | 0 | 0 | 1.15E-10 | 1.11E-08 | UP |
| QKU33644 | alanyl-tRNA synthetase [Tupanvirus deep ocean] | 196.1795 | 0 | 0 | 0 | 0 | 0 | 1.35E-10 | 1.20E-08 | UP |
| CAB4129709 | BaeS Signal transduction histidine kinase [uncultured Caudovirales phage] | 196.1795 | 0 | 0 | 0 | 0 | 0 | 1.35E-10 | 1.20E-08 | UP |
| YP_009602173 | DNA ligase [Escherichia phage ECD7] | 192.7971 | 0 | 0 | 0 | 0 | 0 | 1.41E-10 | 1.21E-08 | UP |
| QJI53211 | hypothetical protein EBPL_00170 [Enterobacter phage EBPL] | 186.0323 | 0 | 0 | 0 | 0 | 0 | 1.54E-10 | 1.22E-08 | UP |
| QHJ78850 | hypothetical protein [Myoviridae sp.] | 186.0323 | 0 | 0 | 0 | 0 | 0 | 1.54E-10 | 1.22E-08 | UP |
| YP_009429951 | Phosphohydrolase incomplete domain [Pandoravirus salinus] | 172.5027 | 0 | 0 | 0 | 0 | 0 | 1.85E-10 | 1.37E-08 | UP |
| YP_009609600 | protector from prophage-induced early lysis [Serratia phage CHI14] | 172.5027 | 0 | 0 | 0 | 0 | 0 | 1.85E-10 | 1.37E-08 | UP |
| QHJ83140 | hypothetical protein [Bacteriophage sp.] | 162.3554 | 0 | 0 | 0 | 0 | 0 | 2.16E-10 | 1.41E-08 | UP |
| NP_955071 | CNPV048 alkaline phosphodiesterase-like protein [Canarypox virus] | 162.3554 | 0 | 0 | 0 | 0 | 0 | 2.16E-10 | 1.41E-08 | UP |
| YP_009174129 | putative alanine racemase [Yellowstone lake mimivirus] | 162.3554 | 0 | 0 | 0 | 0 | 0 | 2.16E-10 | 1.41E-08 | UP |
| CAB4132338 | PinR Site-specific recombinases, DNA invertase Pin homologs [uncultured Caudovirales phage] | 162.3554 | 0 | 0 | 0 | 0 | 0 | 2.16E-10 | 1.41E-08 | UP |
| CAB4167239 | Protein of unknown function DUF932 [uncultured Caudovirales phage] | 155.5906 | 0 | 0 | 0 | 0 | 0 | 2.37E-10 | 1.51E-08 | UP |
| JF974314 | Rhizobium phage RR1-A genomic sequence | 148.8258 | 0 | 0 | 0 | 0 | 0 | 2.65E-10 | 1.59E-08 | UP |
| QHZ54087 | putative metallo-hydrolase YycJ [Paenibacillus phage phiERICV] | 148.8258 | 0 | 0 | 0 | 0 | 0 | 2.65E-10 | 1.59E-08 | UP |
| QKW95371 | DnaC-like protein [Ralstonia phage RPZH6] | 145.4434 | 0 | 0 | 0 | 0 | 0 | 2.81E-10 | 1.65E-08 | UP |
| QGH71839 | hypothetical protein prasa_gp283 [Enterobacter phage prasa_myo] | 138.6786 | 0 | 0 | 0 | 0 | 0 | 3.20E-10 | 1.83E-08 | UP |
| QHJ78869 | hypothetical protein [Myoviridae sp.] | 152.2082 | 0 | 0 | 0 | 0 | 0 | 1.12E-09 | 6.24E-08 | UP |
| DAC81289 | TPA_asm: PolB [Corynactis coral adintovirus] | 2076.797 | 0 | 1485.975 | 0 | 0 | 0 | 7.65E-05 | 0.004159 | UP |
| DAC81267 | TPA_asm: integrase [Bos-associated insect adintovirus 2] | 994.4271 | 0 | 2008.388 | 0 | 0 | 0 | 9.95E-05 | 0.005282 | UP |
| DQ115822 | Cyanobacteria phage AS-1 contig_15 genomic sequence | 0 | 68.81585 | 1393.101 | 0 | 0 | 0 | 0.000289 | 0.014983 | UP |
| DAC81568 | TPA_asm: PolB [Hydra adintovirus] | 0 | 96.34219 | 1102.872 | 0 | 0 | 0 | 0.000383 | 0.019408 | UP |
| FN594518 | Pseudomonas phage phi-2, complete genome, isolated from Pseudomonas fluorescens SBW25 | 0 | 695.0401 | 232.1835 | 0 | 0 | 0 | 0.000548 | 0.027127 | UP |
| YP_009448636 | Cytochrome P450-like protein E-class [Orpheovirus IHUMI-LCC2] | 0 | 474.8294 | 383.1028 | 0 | 0 | 0 | 0.00061 | 0.028975 | UP |
| QMP84056 | DNA-directed RNA polymerase [Pseudomonas phage phiB1_1] | 0 | 454.1846 | 394.712 | 0 | 0 | 0 | 0.000618 | 0.028975 | UP |
| MG747435 | Ralstonia phage RsoM1USA, complete genome | 0 | 412.8951 | 417.9304 | 0 | 0 | 0 | 0.000637 | 0.028975 | UP |
| YP_009789836 | terminase, ATPase subunit [Klebsiella phage 4 LV-2017] | 0 | 412.8951 | 417.9304 | 0 | 0 | 0 | 0.000637 | 0.028975 | UP |
| YP_003345499 | predicted phage lysozyme [Pseudomonas phage phi-2] | 0 | 309.6713 | 441.1487 | 0 | 0 | 0 | 0.000731 | 0.032589 | UP |
| AYJ76167 | putative QueE-like protein [Bacillus phage BSP7] | 0 | 323.4345 | 394.712 | 0 | 0 | 0 | 0.000776 | 0.033923 | UP |
| YP_003345482 | predicted phage DNA Polymerase [Pseudomonas phage phi-2] | 0 | 137.6317 | 545.6313 | 0 | 0 | 0 | 0.00083 | 0.035597 | UP |

Table S2. Differentially expressed virus in 150+ vs 150- comparison

| **VirusID** | **description** | **150+1** | **150+2** | **150+3** | **150-1** | **150-2** | **150-3** | **Pvalue** | **Padj** | **trend** |
| --- | --- | --- | --- | --- | --- | --- | --- | --- | --- | --- |
| YP_009199475 | putative virion structural protein [Pseudomonas phage PaMx74] | 0 | 0 | 0 | 0 | 0 | 617.3749 | 6.56E-12 | 1.81E-08 | DOWN |
| YP_009483076 | Guanylate kinase [Pandoravirus quercus] | 0 | 0 | 0 | 0 | 0 | 389.2146 | 2.06E-11 | 2.43E-08 | DOWN |
| YP_009044608 | DNA polymerase I [Listeria phage LP-083-2] | 0 | 0 | 0 | 0 | 0 | 348.951 | 2.65E-11 | 2.43E-08 | DOWN |
| QGZ18274 | Hsp20 family protein [Pelagibacter phage HTVC115P] | 0 | 121.2162 | 0 | 0 | 0 | 0 | 5.74E-09 | 2.32E-06 | UP |
| QMP19199 | DNA helicase [Pseudomonas phage Persinger] | 0 | 95.77573 | 0 | 0 | 0 | 0 | 6.18E-09 | 2.32E-06 | UP |
| AY080477 | AY080477 Scripps Pier (La Jolla, CA) uncultured virus community uncultured marine virus genomic clone SIO51p10G5R, genomic survey sequence | 0 | 94.27923 | 0 | 0 | 0 | 0 | 6.31E-09 | 2.32E-06 | UP |
| QFG74671 | RNA polymerase Rpb1, domain 5 [Megaviridae environmental sample] | 0 | 91.28624 | 0 | 0 | 0 | 0 | 6.78E-09 | 2.32E-06 | UP |
| NP_958682 | Bbp13 [Bordetella virus BPP1] | 0 | 91.28624 | 0 | 0 | 0 | 0 | 6.78E-09 | 2.32E-06 | UP |
| QKE22869 | hypothetical protein 8P_019 [Pseudomonas phage 8P] | 0 | 86.79675 | 0 | 0 | 0 | 0 | 7.60E-09 | 2.32E-06 | UP |
| YP_002154639 | putative sensor histidine kinase [Feldmannia species virus] | 0 | 110.7407 | 0 | 0 | 0 | 0 | 9.71E-09 | 2.67E-06 | UP |
| NP_690785 | site-specific recombinase for integration and excision [Bacillus phage phi105] | 0 | 92.78274 | 0 | 0 | 0 | 0 | 1.11E-08 | 2.78E-06 | UP |

Table S3. Primer information used in qRT-PCR. All sequences are shown 5’-3’.

| **Gene** | **Forward primer** | **Reverse primer** |
| --- | --- | --- |
| $\beta$*-actin* | AGGTCATCACCATTGGCAAT | GATGTCGACGTCACACACTTCAT |
| *nf-kb* | GGCAGAGTTCGTCAAAGC | AGACGCACAGCCTCCATA |
| *cyp1a1* | AATCCCAGACGGGCTACA | CCGGGCCATAGCACTTAC |
| *cyp1b1* | GCTCAGCTGGTCCATTGATACC | CATCAGCGACAGCAACACAC |
| *tjp2a* | GTGAGGCTAAACACCATTC | TGCTTTGACAGACCCAGA |
| *tjp2b* | TGATGGCTGTTAGGTTTCTG | GGGTAATCTGAGTCTGGGTC |
| *IL-1*$\beta$ | AAGGCTCCGCTCCACATCTCGTA | GTCCATCTCCACCATCTGCGAATCT |
| *IL-6* | TCACGTCATGAACGAGATCC | CCTCTTGCATTTCACCATATCC |
